# Involvement of Noradrenergic Transmission in the PVN on CREB Activation, TORC1 Levels, and Pituitary-Adrenal Axis Activity during Morphine Withdrawal

**DOI:** 10.1371/journal.pone.0031119

**Published:** 2012-02-15

**Authors:** Fátima Martín, Cristina Núñez, M. Teresa Marín, M. Luisa Laorden, Krisztina J. Kovács, M. Victoria Milanés

**Affiliations:** 1 Group of Cellular and Molecular Pharmacology, Department of Pharmacology, School of Medicine, University of Murcia, Murcia, Spain; 2 Instituto Murciano de Investigación Biosanitaria (IMIB), Murcia, Spain; 3 Department of Pharmacy and Pharmaceutical Technology, Faculty of Pharmacy, University of Granada, Granada, Spain; 4 Laboratory of Molecular Neuroendocrinology, Institute of Experimental Medicine, Budapest, Hungary; Emory University, United States of America

## Abstract

Experimental and clinical findings have shown that administration of adrenoceptor antagonists alleviated different aspects of drug withdrawal and dependence. The present study tested the hypothesis that changes in CREB activation and phosphorylated TORC1 levels in the hypothalamic paraventricular nucleus (PVN) after naloxone-precipitated morphine withdrawal as well as the HPA axis activity arises from α_1_- and/or β-adrenoceptor activation. The effects of morphine dependence and withdrawal on CREB phosphorylation (pCREB), phosphorylated TORC1 (pTORC1), and HPA axis response were measured by Western-blot, immunohistochemistry and radioimmunoassay in rats pretreated with prazosin (α_1_-adrenoceptor antagonist) or propranolol (β-adrenoceptor antagonist). In addition, the effects of morphine withdrawal on MHPG (the main NA metabolite at the central nervous system) and NA content and turnover were evaluated by HPLC. We found an increase in MHPG and NA turnover in morphine-withdrawn rats, which were accompanied by increased pCREB immunoreactivity and plasma corticosterone concentrations. Levels of the inactive form of TORC1 (pTORC1) were decreased during withdrawal. Prazosin but not propranolol blocked the rise in pCREB level and the decrease in pTORC1 immunoreactivity. In addition, the HPA axis response to morphine withdrawal was attenuated in prazosin-pretreated rats. Present results suggest that, during acute morphine withdrawal, NA may control the HPA axis activity through CREB activation at the PVN level. We concluded that the combined increase in CREB phosphorylation and decrease in pTORC1 levels might represent, in part, two of the mechanisms of CREB activation at the PVN during morphine withdrawal.

## Introduction

Opiate withdrawal is associated with central noradrenergic neurons hyperactivity, and it has been proposed that noradrenergic afferents to the extended amygdala and to the hypothalamic paraventricular nucleus (PVN) are critically involved in the aversive properties (such as conditioned place aversion) as well as in the somatic symptoms of opiate withdrawal (teeth chattering, piloerection, lacrimation, rinorrhea and ptosis) [1]–[3]. These noradrenergic afferents originate in the nucleus of the solitary tract (NTS) and ventrolateral medulla (VLM) noradrenergic A_2_ and A_1_ cell groups [1], [4].

Clinical and experimental findings have shown that administration of α_1_- and/or β-adrenoceptor antagonists reduced certain aspects of drug withdrawal and dependence, such as the negative emotional status, self administration and relapse [2], [5]–[7]. Furthermore, clonidine, an α_2_-adrenoceptor agonist, has been reported to attenuated withdrawal symptoms in humans and animals [8], [9]. A prime candidate for the central actions of the adrenoceptor antagonists is the PVN, a structure with remarkably dense noradrenergic innervations [10], [11]. Altered neuronal activity has been found in the PVN after naloxone-induced opiate withdrawal, as evidenced by increased activation of the immediate early gene product c-Fos and enhanced hypothalamus-pituitary-adrenocortical (HPA) axis response (as reflected by plasma levels of corticosterone, a marker for the HPA axis activity). These alterations were markedly decreased by systemic adrenoceptor antagonists [2], [12]. Previous works have also shown that lesion of ascending axons of the ventral noradrenergic bundle markedly reduced opiate withdrawal-induced place aversion [1]. Furthermore, pretreatment with adrenoceptor antagonists attenuated heroin self administration in rats [13], suggesting that noradrenergic system may contribute to mechanisms that promote dependence.

On the other hand, morphine dependence exerts long-lasting effects on gene expression [14], [15]. Precipitated morphine withdrawal has shown several indices of cAMP Response Element Binding protein (CREB) function within the PVN, including elevated c-Fos expression in rats [12], [16]. It has been proposed that changes in CREB activity may be important for the development and expression of opioid dependence [17], [18]. Recently, we found increased phosphorylated CREB (pCREB) expression within CRF immunoreactive neurons in the PVN and within tyrosine-hydroxylase (TH)-positive neurons in the nucleus of the solitary tract (NTS) in morphine-withdrawn rats, which paralleled elevation of plasma corticosterone levels [19].

The purpose of the present series of experiments was to test the hypothesis that CREB activation in the PVN and the enhanced response of the HPA axis during naloxone-precipitated morphine withdrawal would arise from the activation of α_1_- and/or β-adrenoceptor. Specifically, the effects of prazosin (α_1_-adrenoceptor antagonist) and propranolol (β-adrenoceptor antagonist), were evaluated for their ability to modulate both CREB activation in the PVN and the pituitary-adrenocortical response to precipitated morphine withdrawal. Phosphorylation of CREB has been used as a marker for the activation of CREB-mediated gene transcription. However, there is recent evidence showing that some extracellular stimuli that cause CREB phosphorylation fail to induce CREB-dependent transcription [20]. These findings suggest that there must be additional CREB co-activators that control the kinetics of CREB-target gene expression. It led to the discovery of a family of coactivators called transducers of regulated CREB activity (TORCs) [21], which facilitate CREB-mediated gene transcription [22]. TORCs are maintained in an inactive state in the cytoplasm as a result of phosphorylation. Different stimuli lead to TORC dephosphorylation and subsequent nuclear accumulation, whereby it can freely associate with CREB. The second aim of the present study was to assess the possibility that the activation of the CREB coactivator, TORC1 in the PVN arises from activation of α_1_- and/or β-adrenoceptor.

## Results

In accordance with previous findings, Student's *t*-test showed that rats receiving long-term morphine treatment had significantly lower weight gain (1.64±2.61 g; t(76) = 6.308; p<0.001; n = 42) than the placebo control group (22.72±1.93 g; n = 36), which might be due to the reduced food intake observed during chronic morphine treatment [23]. The body weight loss after saline or naloxone injection to placebo-pelleted and morphine-dependent rats was recorded as a sign of opiate withdrawal. Two-way ANOVA revealed that chronic pretreatment, acute injection, and the interaction between chronic pretreatment and acute treatment had a significant effect on body weight loss [morphine treatment: F(1,37) = 37.60, p<0.0001; naloxone injection: F(1,37) = 36.91, p<0.0001; interaction: F(1,37) = 16.31, p = 0.0003]. In agreement with our previous results [24], [25], *post hoc* analysis showed (Table 1) that naloxone injection to morphine-dependent animals significantly increased (p<0.001) body weight loss when measured 60 min after injection compared with the placebo-pelleted group also receiving naloxone and with the morphine-treated rats receiving saline. However, administration of naloxone to control rats resulted in no significant changes in body weight loss, compared with control rats receiving saline. In animals pre-treated with prazosin (1 mg/kg i.p.), two-way ANOVA revealed significant effects of chronic pretreatment [F(1,26) = 106.87; p<0.0001] and acute drug injection [F(1,26) = 8.21; p<0.0081]. There was a significant (p<0.01) decrease in body weight loss during morphine withdrawal in animals receiving prazosin 20 min before naloxone injection compared with morphine-withdrawn rats receiving vehicle. In animals pretreated with propranolol (3 mg/kg i.p.), two-way ANOVA showed a significant effect of chronic pretreatment [F(1,25) = 66.53; p<0.0001]. In contrast to prazosin, post hoc test revealed that pretreatment with propranolol did not significantly attenuate the increase in body weight loss in morphine-withdrawn animals compared with morphine-withdrawn rats receiving vehicle instead of propranolol. Neither prazosin nor propranolol modified the weight loss in placebo-pretreated rats compared to placebo-treated rats receiving vehicle.

**Table 1:** Effects of adrenoceptor blockade on body weight loss during morphine withdrawal.

Treatment	Mean (g) ± S.E.M.
pla+sal	0.2±1.3
pla+nx	2.6±0.3
mor+sal	2.6±0.3
mor+nx	14.5±1.2*** +++
pla+veh+nx	3.0±1.3
pla+praz+nx	1.4±0.3
mor+veh+nx	15.8±1.1
mor+praz+nx	11.1±0.9##
pla+veh+nx	2.4±1.0
pla+prop+nx	4.7±0.7
mor+veh+nx	15.8±1.5
mor+prop+nx	13.2±1.6

Rats were treated with pellets of placebo (pla) or morphine (mor) to induce depencence and withdrawal precipitated by naloxone (nx). Other groups of rats received vehicle (veh), prazosin (praz) or propranolol (prop). The weight loss was checked immediately before and 60 min after saline (sal) and naloxone injection. Data show the means ± SEM.

***p<0.001 vs. pla+nx;

+++ p<0.001 vs. mor+sal;

## p<0.01 vs. mor+veh+nx.

### Effects of naloxone-induced morphine withdrawal on NA and MHPG levels and NA turnover in the PVN

**Figure 1:**
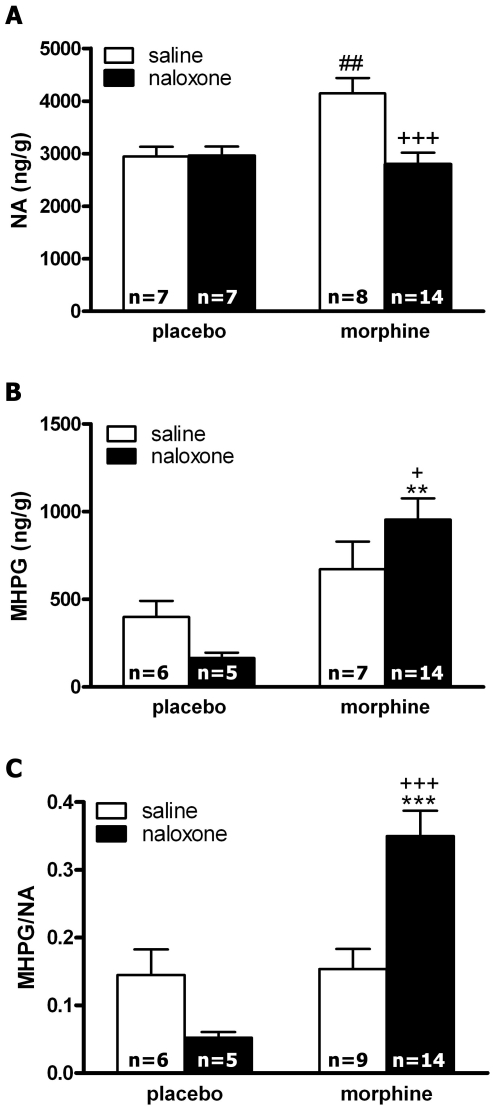
Effects of naloxone-induced morphine withdrawal on NA and MHPG levels at the PVN and on NA turnover (as estimated by the MHPG/NA ratio. Morphine withdrawal increased MHPG production and NA turnover. Data represent the mean ± SEM 60 min after naloxone injection to control pellets- or morphine-treated rats. **p<0.01; ***p<0.001 vs. control pellets (placebo)+naloxone; ^+^p<0.05, ^+++^p<0.001 vs. morphine-treated rats+saline; ^##^ p<0.01 vs. placebo-treated rats+saline.

Fig. 1 summarizes the changes in NA content, 3-Methoxy-4-hydroxyphenylglycol (MHPG; the metabolite of NA at the central nervous system) production and NA turnover (as estimated by the ratio MHPG/NA) after injection of naloxone to control and morphine-dependent rats. The overall ANOVA on NA content in the PVN revealed the main effects of acute treatment [F(1,31) = 10.25; p = 0.0032] and a significant interaction between pretreatment and acute treatment [F(1,31) = 10.49; p = 0.0029]. *Post hoc* analysis indicated that groups rendered dependent on morphine and injected with saline showed significantly (p<0.01) higher levels of NA than the placebo-pelleted groups also injected with saline (Fig. 1A). By contrast, morphine dependent rats receiving naloxone showed a significant (p<0.001) decrease in NA levels 60 min after naloxone injection. The ANOVA for MHPG production showed a significant effect of chronic pretreatment [F(1,28) = 13.61; p = 0.0010]. *Post hoc* analysis showed that the MHPG levels increased significantly (p<0.01) in the naloxone-precipitated morphine withdrawal group, as compared with the placebo-treated group injected with naloxone and with the morphine-dependent rats receiving saline instead naloxone (Fig. 1B). Results for the two-way ANOVA for NA turnover (as revealed by MHPG/NA ratio) in the PVN showed a significant effect of chronic pretreatment [F(1,30) = 14.03; p = 0.0008], and significant interaction between pretreatment and acute treatment [F(1,30) = 12.53; p = 0.0013]. As shown in Fig. 1C, rats rendered dependent on morphine and injected with naloxone showed a significantly higher NA turnover in the PVN than the placebo group injected with naloxone (p<0.001) and than the morphine-pelleted group receiving saline instead naloxone (p<0.001).

### Effects of adrenergic antagonists on morphine withdrawal-induced CREB phosphorylation in the PVN as determined by Western blot and immunohistochemistry

**Figure 2:**
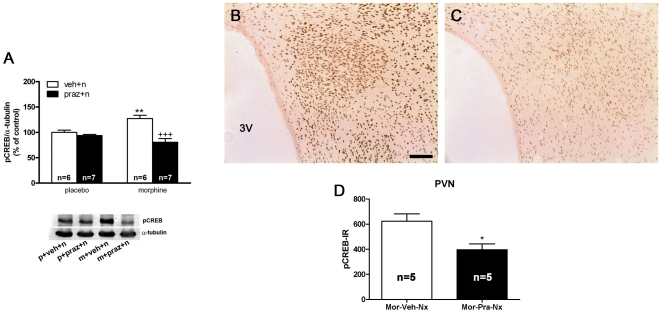
Morphine withdrawal-induced CREB activation in the PVN is dependent on α_1_-adrenoceptor stimulation. Quantitative analysis and representative immunoblots (A) of pCREB in the PVN tissue isolated from placebo or morphine-dependent rats pretreated with prazosin before saline or naloxone injection to control and to morphine-dependent rats. Post hoc analysis revealed that the increase in CREB phosphorylation during morphine withdrawal was blocked by prazosin (1 mg/kg i.p.). Each bar represents mean ± SEM (% of controls); p: placebo pellets; m: morphine pellets; veh: vehicle; n: naloxone; praz: prazosin. **p<0.01 vs control pellets (placebo)+vehicle+naloxone; ^+++^p<0.001 vs. morphine-treated rats+ to control and to morphine-dependent rats. vehicle+naloxone. PVN was also processed for pCREB immunohistochemistry. (B, C) represents immunohistochemical detection of pCREB in the PVN from morphine-treated rats receiving vehicle and naloxone (B) or prazosin plus naloxone (C). 3V: third ventricle. *Scale bar*: 100 µm. D: quantitative analysis of pCREB immunoreactivity the PVN. Data correspond to mean ± SEM. Post hoc analysis revealed a significant decrease in pCREB immunoreactivity in prazosin-pretreated rats. *p<0.05 versus morphine+vehicle+naloxone.

**Figure 3:**
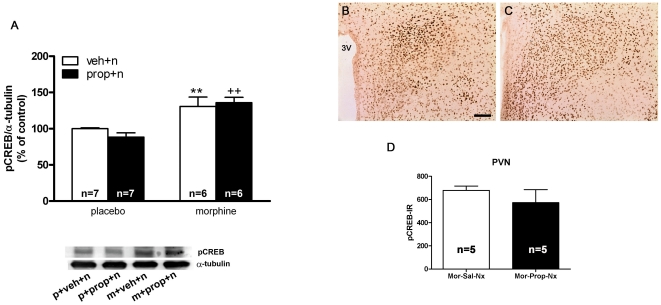
Morphine withdrawal-induced CREB activation in the PVN is not dependent on β-adrenoceptor stimulation. Quantitative analysis and representative immunoblots (A) of pCREB in the PVN tissue isolated from placebo or morphine-dependent rats pretreated with propranolol before saline or naloxone injection to control and to morphine-dependent rats. Post hoc analysis revealed that the increase in CREB phosphorylation during morphine withdrawal was not modified by propranolol (3 mg/kg i.p.). Each bar represents mean ± SEM (% of controls); p: placebo pellets; m: morphine pellets; veh: vehicle; n: naloxone; prop: propranolol. **p<0.01 vs. control pellets (placebo)+naloxone; ^++^p<0.01 vs. placebo-treated rats+propranolol+naloxone. PVN was also processed for pCREB immunohistochemistry. (B, C) represents immunohistochemical detection of pCREB in the PVN from morphine-treated rats receiving vehicle and naloxone (B) or propranolol plus naloxone (C). 3V: third ventricle. *Scale bar:* 100 µm. D: quantitative analysis of pCREB immunoreactivity the PVN. Data correspond to mean ± SEM. Post hoc analysis revealed no significant effects of propranolol pretreatment on pCREB immunoreactivity.

In previous studies, western blot analysis revealed strong activation (phosphorylation) of CREB in the PVN after naloxone injection to morphine-dependent rats, which was dependent on protein kinase C activation [19]. In the present work we examined whether noradrenergic neurotransmission is necessary for the morphine withdrawal-induced CREB phosphorylation. Two-way ANOVA for rats pretreated with the selective α_1_-adrenoceptor antagonist, prazosin revealed that acute prazosin administration [F(1,22) = 23.61; p<0.0001] and the interaction between morphine treatment and prazosin injection [F(1,22) = 13.81; p = 0.0012] had a significant effect on pCREB immunoreactivity in the PVN. As shown in Fig. 2A, Newman-Keuls post hoc test shows that naloxone injection to morphine-dependent rats pretreated with vehicle produced a significant (p<0.01) increase in pCREB levels compared with the placebo-pelleted group also receiving naloxone, which was blocked (p<0.001) in rats pretreated with prazosin 20 min prior naloxone. The results were confirmed by immunohistochemical procedures. As shown in Fig. 2B, high levels of pCREB immunoreactivity were observed in the PVN 60 min after naloxone injection to morphine-dependent rats, whereas the PVN from rats pretreated with prazosin showed discrete staining for pCREB (Fig. 2C). According to the Western-blot analysis, there was a decrease (t(8) = 3.035; p<0.05) in pCREB immunoreactivity 60 min after naloxone administration to morphine-dependent rats pretreated with prazosin (Fig. 3D). Two-way ANOVA for rats pretreated with the β-adrenoceptor antagonist, propranolol revealed significant effect of pretreatment [F(1,22) = 26.44; p<0.0001] on pCREB immunoreactivity in the PVN. Newman-Keuls post hoc test shows that administration of propranolol 20 min prior naloxone injection produced a similar increase (p<0.01) in pCREB levels than that seen in morphine dependent rats pretreated with vehicle instead propranolol (Fig. 3A). These results were also confirmed by immunohistochemical procedures. As shown in Fig. 3B, C, the PVN from rats pretreated with propranolol shows similar staining for pCREB than the PVN from rats receiving vehicle instead propranolol. No significant differences (t(8) = 1.060) were seen between the morphine-dependent rats receiving vehicle plus naloxone and those injected with propranolol plus naloxone (Fig. 3D).

### Induction of CREB phosphorylation in CRF-positive neurons in the PVN is attenuated by prazosin

**Figure 4:**
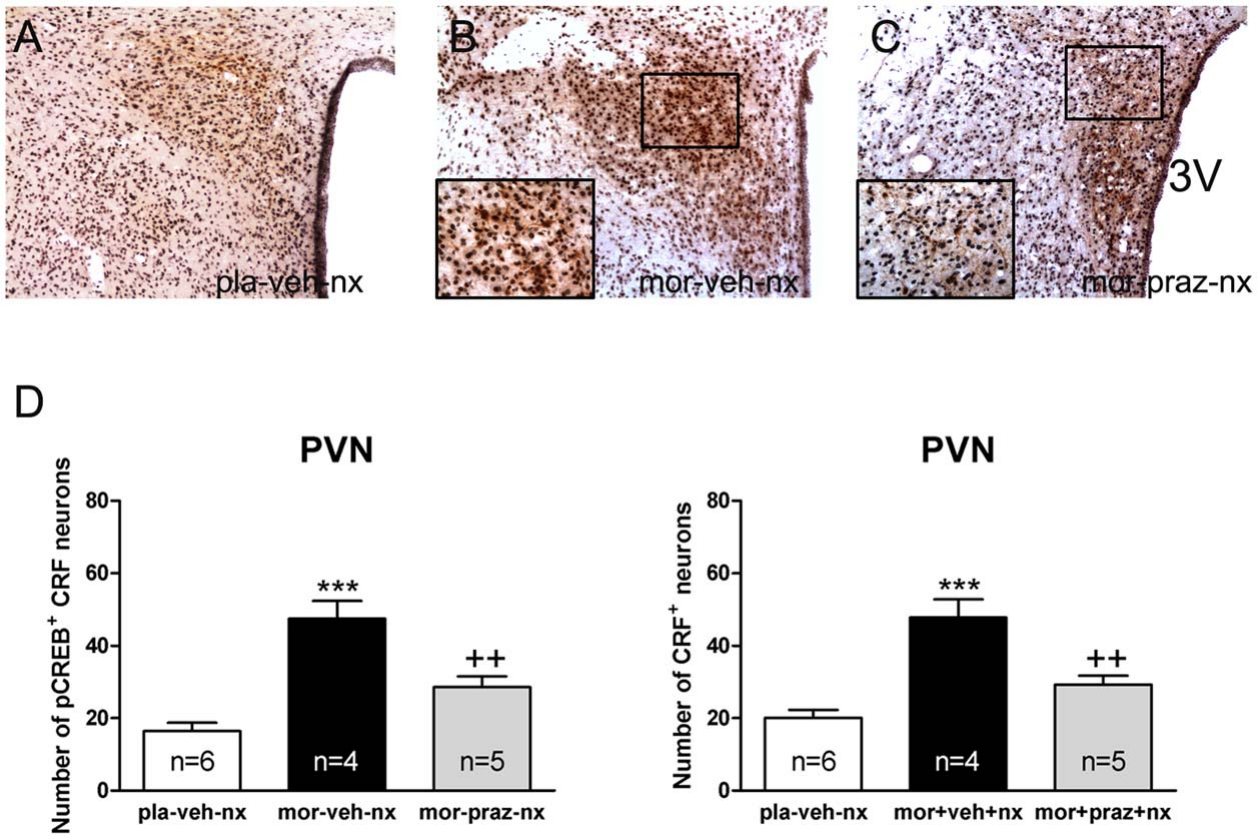
Increased pCREB into CRF neurons after naloxone-induced morphine withdrawal is α-1 adrenoceptor dependent. PVN tissue isolated from placebo or morphine-dependent rats pretreated with vehicle or prazosin before naloxone injection was processed for pCREB and CRF double-label immunohistochemistry. Top panels (A–C) represent immunohistochemical detection of pCREB into CRF neurons after the different treatments. Low and high magnifications images show pCREB-positive (blue-black)/CRF-positive (brown) neurons in the PVN. *Scale bar:* 100 µm (low magnification); 20 µm (high magnification). 3V, third ventricle. Bottom panels (D) show quantitative analysis of pCREB-positive/CRF-positive and total CRF-positive (with or without pCREB) neurons in the PVN. Data shown are means ± SEM. *Post hoc* test revealed a higher number of pCREB-positive nuclei in CRF immunoreactive neurons after naloxone-induced morphine withdrawal. This increase was antagonized in prazosin-pretreated rats. The increase in number of CRF-positive neurons during morphine withdrawal was also blocked by prazosin. ***p<0.001 versus placebo (pla)+vehicle (veh)+naloxone (nx); ^++^p<0.01 versus mor+veh+nx.

To explore the specificity of the decrease of pCREB levels observed in the parvocellular part of the PVN during morphine withdrawal in animals pretreated with prazosin, sections from different treatment were immunohistochemically double-labeled for pCREB and CRF (Figure 4 A–C). ANOVA revealed significant differences in the number of CRF neurons expressing pCREB in rats pretreated with prazosin [F(2,14) = 21,69; p<0.001]. As shown in Figure 4D (left panel), *post hoc* comparisons showed a significant (p<0.01) decrease in the number of CRF neurons containing pCREB after naloxone-induced morphine withdrawal in prazosin-pretreated rats compared with those receiving vehicle instead of prazosin. Additionally, ANOVA also revealed significant differences [F(2,14) = 19,28; p<0.001] in CRF immunoreactivity in rats pretreated with prazosin. As shown in Figure 4D (right panel), there was a significant (p<0.01) decrease in the total number of CRF neurons after naloxone-induced morphine withdrawal in prazosin-pretreated rats.

### Effects of prazosin on pTORC1 lower expression during morphine withdrawal

**Figure 5:**
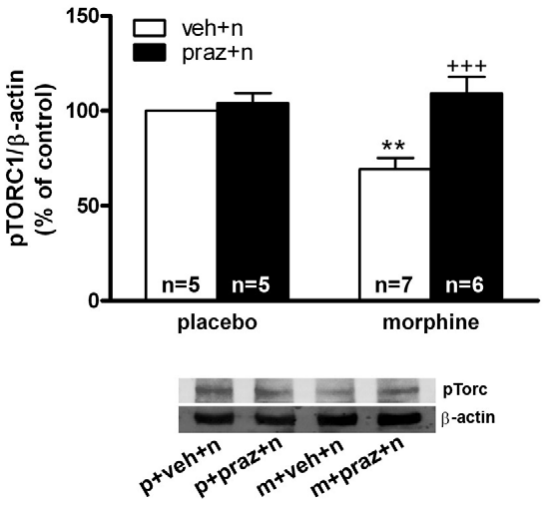
Noradrenergic activity is required for morphine withdrawal-induced TORC1 activation in the hypothalamic PVN. Quantitative analysis and representative immunoblots of phosphorylated TORC 1 in the PVN tissue isolated from placebo or morphine-dependent rats pretreated with vehicle or prazosin before saline or naloxone injection to control and to morphine-dependent rats. Post hoc analysis revealed that the decrease in TORC phosphorylation induced by morphine withdrawal was reversed by prazosin. Each bar represents mean ± SEM (% of controls); p: placebo pellets; m: morphine pellets; veh: vehicle; n: naloxone; praz: prazosin. **p<0.01 vs. control pellets (placebo)+vehicle+naloxone; ^+++^p<0.001 vs. morphine-treated rats+vehicle+naloxone.

Two-way ANOVA revealed a significant effect of acute injection [F(1,19) = 11.89; p = 0.0027], and an interaction between chronic pretreatment and acute treatment [F(1,19) = 7.87; p = 0.0113] on pTORC1 levels at the PVN. As shown in Fig. 5, Western blot analysis revealed a significant (p<0.01) decrease for pTORC1 immunoreactivity in morphine-withdrawn rats compared with the control group receiving naloxone. To determine the ability of the α_1_-adrenoceptor blockade on pTORC1 expression, control- and morphine- treated rats were pretreated with prazosin 20 min before saline or naloxone injection. Post hoc analysis showed that prazosin reverted (p<0.001) the decreased pTORC1 levels observed during morphine withdrawal.

### Effects of adrenergic antagonists on morphine withdrawal-induced HPA axis activation

**Figure 6:**
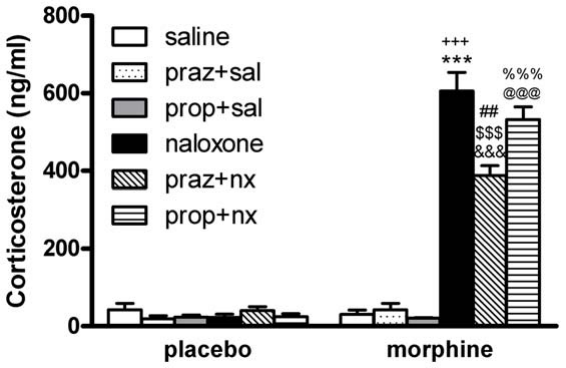
Hypothalamus-pituitary-adrenal (HPA) axis activation during morphine withdrawal is attenuated by α_1_- but not β-adrenoceptor blockade. Placebo and morphine-dependent rats were pretreated with prazosin or propranolol and plasma levels of corticosterone (a marker of HPA axis activity) were determined 60 min after naloxone injection. Praz: prazosin; prop; propranolol; sal: saline; nx: naloxone. Each bar represents mean ± SEM. Post hoc analysis revealed a significant increase in plasma corticosterone concentration after naloxone-induced morphine withdrawal, which was attenuated in prazosin- but not in propranolol-pretreated rats. ***p<0.001 versus placebo+naloxone; ^+++^p<0.001 versus morphine+saline; ^&&&^p<0.001 versus placebo+prazosin+naloxone; ^$$$^p<0.001 versus morphine+prazosin+saline; ^##^p<0.01 versus morphine+naloxone; @@@p<0.001 versus placebo+propranolol+naloxone; ^%%%^p<0.001 versus morphine+propranolol+saline.

We measured plasma corticosterone concentrations (as HPA axis activation marker) in blood samples obtained from morphine-dependent or control rats 60 min after injection of saline or naloxone. Two-way ANOVA for corticosterone revealed a significant effect of chronic pretreatment [F(1,16) = 111.58; p<0.0001], significant effect of acute drug administration [F(1,16) = 117.24; p<0.0001] and significant interaction between acute treatment and chronic pretreatment [F(1,16) = 127.58; p<0.0001]. As shown in Fig. 6A, in morphine-withdrawn rats plasma corticosterone levels increased significantly (p<0.001) compared with those observed in the placebo group also receiving naloxone and the morphine dependent rats receiving saline. Two-way ANOVA for corticosterone in rats receiving prazosin revealed a significant effect of acute injection [F(1,17) = 106.56; p<0.0001], chronic pretreatment [F(1,17) = 108.78; p = 0.0001], and significant interaction between acute and chronic treatment [F(1,17) = 83.10; p<0.0001]. As shown in Fig. 6B, although morphine-withdrawn rats pretreated with prazosin showed elevated (p<0.001) plasma corticosterone levels compared with its control group, a reduction (p<0.01; Dunnet test) in corticosterone levels was observed in morphine pretreated rats injected with prazosin before naloxone compared with morphine-withdrawn rats. Two-way ANOVA for corticosterone in rats receiving propranolol revealed a significant effect of acute injection [F(1,17) = 165.49; p<0.0001], chronic pretreatment [F(1,17) = 160.72; p = 0.0001], and significant interaction between acute and chronic pretreatment [F(1,17) = 164.21; p<0.0001]. As shown in Fig. 6C, in morphine-withdrawn rats administered propranolol, the plasma corticosterone levels increased significantly (p<0.001). By contrast to prazosin pretreatment, pretreatment with propranolol did not modify the morphine withdrawal-induced increase in corticosterone levels. Neither prazosin nor propranolol induced any significant modification in plasma levels of corticosterone in control rats receiving saline or naloxone or in morphine-pretreated rats receiving saline.

## Discussion

For many years, studies have focused on the role of dopaminergic reward system in drug abuse. However, although the role of NA in stress is well known, its involvement in drug addiction has received less attention. It has been demonstrated that opiate withdrawal results in marked activity of central noradrenergic neurons [1], [26]. Thus, several biochemical and electrophysiological changes induced by opiate abstinence have been reported, consisting of an increase in firing rate response by application of opiate antagonists after chronic morphine treatment [27], [28]. Furthermore, NA caused a marked increase in the frequency of postsynaptic potentials of the parvocellular neurons of the PVN [29]. There is also evidence that increased NA is involved in various aspects of the withdrawal response [23], [26].

The PVN, a component of the HPA stress axis, has a high density of noradrenergic inputs [1], [30] and is anatomically connected with other brain areas implicated in drug abuse, such as the extended amygdala (the brain stress system) and the NTS-A_2_. We therefore hypothesized that the HPA axis may be an important site for the actions of NA during withdrawal. Previous studies from our group indicate that NA turnover is increased in the PVN 30 min after naloxone administration to morphine-dependent rats [2]. Present findings show that morphine withdrawal also enhances noradrenergic activity in the PVN at 60 min time-point, as revealed by increased MHPG production and NA turnover in this nucleus, as Fig. 1 depicts. These effects have been shown to be accompanied by increased CRF hnRNA, TH mRNA expression and tyrosine-hydroxylase (TH) enzymatic activity in the PVN and are induced via a mechanism involving phosphorylation of TH at Ser31 [2], [23]. The present study was focused on the impact of noradrenergic modulation in the context of withdrawal-induced CREB phosphorylation and HPA axis activation that is observed in morphine-withdrawn rats.

As reported recently [19], the data depicted in Fig. 2 indicate that naloxone-induced morphine withdrawal produced robust CREB activation in the hypothalamic PVN. These effects of morphine abstinence are mediated through the activation (phosphorylation) of CREB, but not through the up-regulation of its expression in the PVN, as previously shown by Martín et al. [18], [19]. CREB regulates the transcription of over 10,000 genes, including those implicated in stress and addiction, such as CRF [31]. The present work showed that the increase in pCREB immunoreactivity co-localized with CRF neurons of the parvocellular part of the PVN (Fig. 4), consistent with the morphine withdrawal-induced the transcriptional regulation of CRF in the PVN. Thus, using probes complementary to intronic sequences of the gene encoding CRF in the parvocellular neurosecretory neurons of the PVN, we had found robust increases in the precursor mRNA (hnRNA) for CRF in morphine-dependent rats during naloxone-precipitated morphine withdrawal [16]. In addition, previous findings showed that the induction of c-Fos expression that occurs during morphine withdrawal occurs predominantly in hnRNA CRF-expressing neurons of the parvocellular part of the PVN, consistent with transcriptional regulation of CRF neurons by morphine withdrawal [16]. Taken together, present results might suggest that activation of CREB could contribute to the increased CRF gene-transcription during morphine withdrawal. Supporting this hypothesis are previous findings indicating that CREB is a potent activator of CRF transcription [20], [32]. Furthermore, Itoi et al. [31] showed that injection of antisense oligodeoxynucleotides to CREB blocked the increase in CRF mRNA caused by stress and drug exposure [33]. According to these findings, in the present study we have shown that morphine withdrawal increased CRF immunoreactivity in the PVN, as Fig. 4 depicts. The increase in number of CRF-positive neurons could be the results from an increase in the synthesis of the peptide then an increase in the peptide content in the CRF perikaria of the PVN. Although indirectly, all these results might suggest that activation of CREB could contribute to increased transcription of CRF gene during morphine withdrawal.

In the present study, the effects of α_1_- (prazosin) and β- (propranolol) adrenoceptor antagonists were evaluated for their ability to modify CREB phosphorylation, CRF immunoreactivity and corticosterone release in morphine-dependent rats. Present findings clearly show that, at the PVN level, only the α_1_-adrenoceptor can stimulate CREB phosphorylation, since prazosin but not propranolol significantly decreased morphine withdrawal-induced CREB phosphorylation (Figs. 2, 3). Furthermore, noradrenergic α_1_ receptor blockade by prazosin significantly attenuated the morphine withdrawal-induced CREB activation into CRF-positive neurons (Fig. 4). We showed that this response was associated with a reduction of both CRF-containing neurons and corticosterone release, as Figs. 4 and 6 depict. These findings suggest that the ability of morphine withdrawal to stimulate CREB activation and the stress axis activity is under control of noradrenergic system via α_1_-adrenoceptor stimulation. This is in accordance with several reports describing the modulatory action of the noradrenergic system on the hypothalamic stress axis. Indeed, NA neurons arising from the NTS-A_2_ provide excitatory inputs to the CRF neurons in the PVN, and activation of these neurons during precipitated morphine withdrawal or during stress was blocked by prazosin [12], [34]. Since NA was found to stimulate CREB phosphorylation [35], [36], and considering that CREB phosphorylation is critical for CRF transcription, it is reasonable to hypothesize that inhibition of morphine withdrawal-induced CREB activation by prazosin may be responsible for the inhibitory effect of this adrenoceptor antagonist on HPA axis activity, whereas the β-adrenoceptor seems not to be involved in those actions.

Electrical stimulation of the ventral ascending noradrenergic bundle and intracerebroventricular injection of NA, increase pituitary-portal plasma levels of CRF [37]. Moreover, NA injection directly into the PVN has a similar effect, which was prevented by α_1_ but not β-adrenoceptor antagonists [38]. Taken together, these results would suggest a positive correlation between noradrenergic terminals innervating the PVN and HPA axis activity. In addition to the proposed direct effects of NA on adrenoceptor located on the CRF neurons, it has been shown that NA can also influence the activity of the HPA axis through activation of adrenergic receptors located on the bed nucleus of the stria terminalis (BNST; [39]) in response to stress. Daftary et al. [29] reported that CRF release may be also evoked through intrahypothalamic glutamatergic interneurons expressing α_1_-adrenoceptors, indicating the complexity of the interaction between noradrenergic system and CRF neurons.

CREB is classically considered to be the mediator of c-AMP/PKA-mediated effects. According to the conventional model, cAMP activates PKA, leading to sequential phosphorylation of CREB, binding of phospho-CREB to the c-AMP-Response Element (CRE) in the CRF promoter and activation of transcription [40]. According to our results, it has been shown that noradrenergic neurons stimulate CRF cells via α_1_-adrenoceptors and hence the HPA axis [41]–[43]. The α_1_ receptor is coupled to phospholipase C/PKC signal transduction pathway. Thus, it is possible that stimulation of phospholipids signaling by NA would lead to CREB phosphorylation and subsequent activation of the CRF neurons in the PVN from morphine-withdrawn rats. Supporting this possibility, PKC antagonists prevented the observed morphine withdrawal-induced CREB phosphorylation into CRF neurons in the rat PVN [19]. In addition, activation of calcium phospholipids-dependent pathways by the phorbol ester PMA also activated CREB phosphorylation in hypothalamic neurons [20]. According to all these findings, the results of the present study strongly suggest the relevance of α_1_-adrenoceptor in mediating the CREB phosphorylation that was seen after naloxone-induced morphine withdrawal. In addition, our findings support a facilitatory influence of NA on morphine withdrawal-induced HPA activation. However, since prazosin attenuated, but did not block the HPA axis response to morphine withdrawal, others receptor systems may be activated in addition to α_1_-adrenoceptors, such as CRF2 receptors and orexin receptors [44], [45]. An additional explanation for the present findings is that, although CRF is thought to be the major secretagogue in stimulating ACTH secretion, AVP and other factors also play a role [46], [47].

Previous studies suggest that the phosphorylation site of CREB is a convergence point for multiple kinases and acts as a molecular switch for controlling gene activation kinetics [48]. CREB activity can also be regulated by the new family of transcriptional coactivators, TORCs [49]. It has been recently shown that CREB is essential but not sufficient for activation of CRF transcription, suggesting that translocation of TORCs to the nucleus is required for CRF transcription by acting as a CREB coactivator [50]. TORCs phosphorylation by specific kinases increases its affinity with the scaffolding protein 14-3-3, thus preventing nuclear translocation [51]. The present study focused on investigating the effects of morphine withdrawal and adrenoceptor blockade on phosphorylated TORC1 (pTORC1) levels. Our results show that morphine withdrawal produced a decrease of pTORC1, the inactive form of this CREB coactivator (Fig. 5), which suggests that TORC1 was dephosphorylated (activated) in response to morphine abstinence. The mechanism regulating TORCs activation is under current investigation [50]. Our findings also show that pretreatment with prazosin antagonized the morphine withdrawal-induced decreased of pTORC1 levels in the PVN. Therefore, all this evidence might indicate that morphine withdrawal-induced activation of TORC1 would require the activation of α_1_- adrenoceptor, which suggests that phospholipids-dependent pathways might be involved in TORC1 activation, providing a mechanism by which morphine withdrawal and α_1_- agonist induce a stimulatory effect on CRF neurons.

It has been shown that the use of ligands targeting noradrenergic receptor subtypes can attenuate both the physical and motivational components of enhanced drug ingestion that has been observed in opiate-, alcohol-, and nicotine-dependent animals and humans [6], [7], [13], [52], [53]. The PVN appears to be a very important site of action for the α_1_-ligand-mediated effects on feeding [13]. The observation that prazosin but not propranolol attenuated the reduction in weight loss during morphine withdrawal would indicates that noradrenergic pathways may participate in a subset of somatic withdrawal signs through stimulation of α_1_-adrenoceptor subtype, as has been previously shown [2].

In summary, our results suggest that NA and α_1_-adrenoceptors may control the HPA axis activity through CREB activation at the PVN during acute morphine withdrawal. The combination of CREB phosphorylation and in pTORC1 dephosphorylation (activation) might represent, in part, two of the mechanisms of CREB activation at the PVN during morphine withdrawal.

## Materials and Methods

### Animals

Male Sprague-Dawley rats (220–240 g; Harlan, Barcelona, Spain) were housed two-to-three per cage in a room with controlled temperature (22±2°C) and humidity (50±10%), with free access to water and food. Animals were adapted to a standard 12-h light-dark cycle for 7 days before the beginning of the experiments. All surgical and experimental procedures were performed in accordance with the European Communities Council Directive of 24 November 1986 (86/609/EEC) and the local Committees for animal research (REGA ES300305440012). The study was approved by the University of Murcia bioethics committee (RD 1201/2005) and Ministerio de Ciencia y Tecnología (SAF2009-07178), Spain.

### Drug treatment and experimental procedure

Groups of rats were rendered dependent on morphine by s.c. implantation of morphine base pellets (75 mg), one on day 1, two on day 3 and three on day 5, under light ether anesthesia. Control animals were implanted with placebo pellets containing lactose instead of morphine on the same time schedule. This morphine treatment paradigm has been shown to produce profound states of tolerance and dependence and to result in characteristic biochemical adaptations within the paraventricular nucleus and behavioral alterations [23], [54]. On day 8, when rats were morphine dependent, animals were injected i.p. with vehicle, prazosin (1 mg/kg) or propranolol (3 mg/kg) and 20 min later received saline s.c. or naloxone (2 mg/kg s.c.). On the basis of our initial experiments of prazosin-induced inhibition of behavioral signs of morphine withdrawal and HPA axis activity [2], [12] 1 mg/kg dose of prazosin was chosen for our experiments. Dose of propranolol was selected on the basis of previous findings [12]. The weight gain of the rats was checked during treatment to ensure that the morphine was liberated correctly from the pellets because it is known that chronic morphine treatment induces a decrease in body weight gain due to lower caloric intake [55]. In addition, the day of experiment weight loss was determined as the difference between the weight determined immediately before saline or naloxone injection and a second determination made 60 min later, immediately before killing.

### Western blot analysis

Sixty min after administration of naloxone or saline, rats were killed by decapitation. The hypothalamic tissue containing the PVN was dissected according to the technique of Palkovits [56]. PVN samples were placed in homogenization buffer [23], homogenized and sonicated for 30 s before centrifugation at 6,000× g for 10 min at 4°C. Samples containing 40 µg of protein were loaded on a 10% SDS/polyacrylamide gel, electrophoresed and transferred onto polyvinylidene difluoride (PVDF) membranes (Millipore, Bedford, MA, USA). Western analysis was performed with the following primary antibodies: 1∶750 polyclonal anti-phospho CREB-123-136 (pCREB; Millipore, Temecula, CA); 1∶375 polyclonal anti-phospho-TORC1 (pTORC1; Cell Signaling); 1∶5000 polyclonal anti-α-tubulin; and 1∶1000 polyclonal β-actin (Cell Signaling). After extensive washing with TBST, the membranes were incubated with peroxidase-labeled secondary antibodies. We used α-tubulin or β-actin (depending on the molecular weight of the protein measured) as our loading controls for all the experiments. Before re-probing, blots were stripped by incubation with stripping buffer (glycine 25 mM and SDS 1%, pH 2) for 1 h at 37°C.

Blots were subsequently reblocked and probed with anti α-tubulin or β-actin. Quantification of immunoreactivity corresponding to pCREB (43 kDa), pTORC1 (82 kDa), α-tubulin (52 kDa) and β-actin (45 kDa) bands was carried out by densitometry. The integrated optical density of the bands was corrected by subtraction of the background values. The ratios of pCREB/α-tubulin and PTORC1/β-actin were calculated and expressed as a percentage of the average of controls in each blot.

### Immunohistochemical detection of pCREB

Sixty min after naloxone or saline injections, rats were deeply anesthetized with pentobarbital (100 mg/kg ip) and quickly perfused through the ascending aorta with saline followed by ice-cold fixative. Brains were post-fixed in the fixative for 3 h and then placed in PBS containing 10% sucrose overnight. Series of 30 µm frontal sections were cut on freezing microtome, collected in cryoprotectant and stored at −20°C until processing. After blocking with H_2_O_2_ and normal goat serum, sections were then incubated for 60 h at 4°C with a rabbit anti-pCREB antibody (Upstate; 1∶750). This was followed by application of a biotinylated anti-rabbit IgG (Vector Laboratories, Burlingame, CA, USA), and then with the avidin–biotin complex. Visualization of the antigen–antibody reaction sites was performed using 3, 3′-diaminobenzidine (DAB, Sigma). Sections were mounted onto chrome-alumn gelatine coated slides, dehydrated through graded alcohols, cleared in xylene and cover slipped with dibutylphtalate (DPX).

### Double-labeling immunohistochemistry of pCREB-immunoreactive nuclei and CRF-positive neurons

For pCREB and CRF double-label immunohistochemistry, tissue sections from each rat in each treatment group were processed as follows: pCREB was developed with DAB intensified with nickel, and CRF revealed with DAB. pCREB immunohistochemistry was performed as described above, and pCREB antibody-peroxidase complex was visualized by using a mixture of NiSO_4_.6H_2_O (33.2 mg/ml), DAB (0.033%) and 0.014% H_2_O_2_ in 0.175 M sodium acetate solution (pH 7.5). Sections were then incubated for 60 h at 4°C with rabbit anti-CRF antibody (1∶1000 in PBS containing 2% goat serum and 0.5% Triton-X-100; a generous gift from Wylie Vale, The Salk Institute, La Jolla, CA, USA). A biotinylated anti-rabbit IgG (diluted 1∶200) for 1 h was used as a secondary antibody. The CRF antibody-peroxidase complex was stained in 0.033% DAB and 0.014% H_2_O_2_ in 0.05 M Tris-HCl buffer.

### Quantification of pCREB immunoreactivity

pCREB immunostaining within section of the PVN was quantified bilaterally for each rat and for all treatment groups by an observer blinded to the treatment protocol. The density of pCREB-like immunoreactivity was determined using a computer-assisted image analysis system (QWIN, Leica, Madrid, Spain). This system consists of a light microscope (DM4000B; Leica) connected to a video camera (DFC290, Leica) and the image analysis computer.

### Quantification of pCREB-positive/CRF-positive neurons

pCREB-positive CRF cells were identified as cells with brown cytosolic deposits for CRF-positive staining and blue/dark nuclear staining for pCREB. A square field (195 µm) was superimposed upon captured image to use as reference area. The number of double-labeled pCREB neurons observed bilaterally was counted in three to four sections from each animal in the PVN. The total number of CRF cells (with or without a visible nucleus) was also counted.

### HPLC

NA and its metabolite in the central nervous system, MHPG, were determined by HPLC with electrochemical detection as described previously [57], frozen in liquid nitrogen, weighed, placed in perchloric acid (0.1 M), homogenized and centrifuged and the supernatants taken for analysis and filtered through 0.22 mm GV (Millipore). Ten mL of each sample were injected into a 5-mm C18 reversed-phase column (Waters, Milford, MA, USA) through a Rheodyne syringe loading injector (Waters). Electrochemical detection was accomplished with an electrochemical detector (Waters 2465). NA and MHPG were quantified by reference to calibration curves run at the beginning and the end of each series of assays. The content of NA and MHPG in the PVN was expressed as ng·g^−1^ wet weight of tissue. The NA turnover was determined as the NA ratio, which was calculated as: NA ratio = MHPG/NA.

### Radioimmunoassay

Sixty min after saline or naloxone injection, rats were decapitated. Plasma levels of corticosterone were measured by commercially available kits for rats (^125^I-corticosterone RIA; MP Biomedicals, Orangeburg, NY). The sensitivity of the assay was 7.7 ng.mL^−1^.

### Materials

Pellets of morphine (75 mg morphine base/pellet; Alcaliber Labs., Madrid, Spain) or lactose (placebo) were prepared by the Department of Pharmacy and Pharmaceutics Technology (School of Pharmacy, Granada, Spain); naloxone HCl, prazosin HCl and DL-propranolol HCl were purchased from Sigma Chemical Co. (St Louis, MO). Naloxone HCl and propranolol were dissolved in sterile 0.9% NaCl (saline); prazosin was dissolved in sterile distilled water and administered in volumes of 0.1 ml/100 g body weight. Phosphatase inhibitor Cocktail Set (Calbiochem, Germany); protease inhibitors (Boehringer Mannheim, Germany). HPLC reagents were purchased from Sigma.

### Statistical analysis

Data are presented as mean ± S.E.M. Data were analyzed using one- or two-way analysis of variance (ANOVA) followed by a *post hoc* Newman–Keuls test. Student's *t*-test was used when comparisons were restricted to two experimental groups. Differences with a P-value <0.05 were considered significant.
